# How socioeconomic status shapes health outcomes following severe falls: a cross-sectional analysis

**DOI:** 10.1186/s12877-025-06198-9

**Published:** 2025-07-16

**Authors:** Elisa-Marie Speckmann, Lars Schwettmann, Laura Himmelmann, Tania Zieschang, Tim Stuckenschneider

**Affiliations:** 1 Department for Health Services Research, Geriatric Medicine, School of Medicine and Health Services, Carl von Ossietzky University Oldenburg, Oldenburg, Germany; 2 Department for Health Services Research, Health Economics/School of Medicine and Health Services, Carl von Ossietzky University Oldenburg, Oldenburg, Germany

**Keywords:** Emergency department, Falls, Health, Older adults, Socioeconomic status.

## Abstract

**Objective:**

Falls are a major health concern, leading to severe injuries, fatalities, and increased risk of future falls. Severe falls, requiring medical care, necessitate targeted interventions. Socioeconomic status (SES), measured by income and education, influences health outcomes, with lower SES linked to greater multimorbidity and reduced physical activity. While SES broadly impacts fall risk, limited research explores its effects following severe falls. Therefore, this study aims to evaluate the associations between socioeconomic status (SES) and health outcomes in older adults following a fall with presentation to the emergency department (ED).

**Methods:**

We analyzed data from the SeFallED study, comprising individuals over the age of 60, who presented to the ED following a recent fall, without requiring subsequent hospitalization. Income and education were used to characterize SES. Health outcomes include mental and functional performance, physical activity, mental well-being, and utilization of the health system. Linear and logistic regression models, adjusted for age and sex, were used to assess associations with health outcomes, incorporating multicollinearity checks, residual diagnostics, and bias-corrected bootstrapping to ensure robustness and reproducibility.

**Results:**

In this cross-sectional study of 172 older adults (median age 74 years) following a severe fall, education was positively associated with functional and mental outcomes, including Activities of Daily Living (ADLs), cognitive status, hand grip strength, and physical performance (*p* < .001). Regression analysis, adjusting for age and sex, revealed that higher education was significantly associated with better ADLs, cognitive status, hand grip strength, and physical performance, while income showed no significant associations with these outcomes.

**Conclusion:**

This study revealed that the associations with mental and functional health outcomes in older adults following a severe fall were the strongest for education, rather than income. Higher education was associated with better performance. Income showed limited associations but was negatively correlated with concerns about falling. Age and sex were critical covariates, influencing key health outcomes. These findings provide insights that may be relevant for future research and considerations in studies involving older adults presenting to EDs.

**Trial registration:**

DRKS00025949 https://drks.de/search/en/trial/DRKS00025949, Registered on 4th November, 2021.

**Supplementary Information:**

The online version contains supplementary material available at 10.1186/s12877-025-06198-9.

## Background

Falls are a significant concern as they can result in deaths [1], or severe injuries such as fractures [2], and may lead to concerns about falling [3]. Both, concerns about falling and prior falls, along with age and sex, are key risk factors for future falls [4]. In Germany, 25% of individuals over the age of 65 experience at least one fall per year, and 13% fall twice or more [5]. This is in line with the global prevalence of falls of 26.5% among older adults, as reported in a recent meta-analysis [6] and reflects the comparable burden observed across Western Europe [2]. The World Guidelines for Falls Prevention [7] classify severe falls as those requiring medical care or visits to the emergency department (ED). In Germany, within 12 months, 3.0% of women and 1.8% of men over 70 experience falls requiring medical attention [8]. Individuals, who experience severe falls, are at high risk for further falls and are in need for close follow-ups, risk assessments, and targeted interventions [7].

The fall risk of older adults may also be associated with the socioeconomic status (SES) [9], a multidimensional construct that reflects social and economic circumstances [10], which in turn influence health outcomes. While SES is not uniformly defined and its operationalization can vary across studies [10], it is commonly assessed using indicators such as income, education, and occupation [11]. Among older populations income and education are the most frequently used SES indicators [12]. Other terms such as social background [10], or socioeconomic position [13] are sometimes used interchangeably to SES, though they may carry distinct conceptual meanings.

Individuals with lower SES are at an increased risk of multimorbidity [14] and suffer from a greater decline in variables related to aging, such as physical activity [15] than individuals with a higher SES. Data from Germany shows that individuals with lower SES have a lower life expectancy [16] and report a lower subjective health status [17]. Understanding how the SES is linked to individual health is crucial for mitigating inequalities [9] that may affect both fall risk as well as recovery and secondary prevention following a severe fall.

Health selection and social causation are two theories describing relationships between health and socioeconomic factors [18]. Health selection describes the theory, which assumes that health may influence the SES [18]. In contrast, social causation posits that SES affects health outcomes, which may be more applicable in older European adults, and thus, more likely to shape long term trajectories [18]. Older adults who experience a severe fall and face socioeconomic deprivation represent a highly vulnerable but understudied group, potentially at greater risk of future health decline. Although several studies have explored the relationship between falls and SES factors, most of them focus on general older adult populations or compare individuals with and without falls [9, 19–21]. However, limited research has specifically targeted older adults who experienced a severe fall [22, 23] – an event often associated with higher risk for long-term health decline [7]. This study aims to close this gap by focusing on this vulnerable subgroup to better understand the association between SES and various health outcomes post-fall. Within this cross-sectional study we examined the associations between income and education and outcomes such as mental and functional performance, physical activity, mental well-being, and health care utilization in a group of older adults following an acute severe fall.

## Methods

### Study population

Data for this cross-sectional analysis were derived from the ongoing SeFallED study (Sentinel Fall Presenting to the Emergency Department), initiated in 2021 in Oldenburg, Germany. SeFallED is an observational mixed-methods study aiming to identify trajectories of functional decline in adults aged 60 years and older who presented to the ED of the Klinikum Oldenburg or the Evangelisches Krankenhaus Oldenburg following a fall with discharge within 72 h. If informed consent was provided, participants were followed-up for 24 months. Exclusion criteria included: (1) life expectancy of less than 3 months, (2) unstable medical, neurological or psychiatric condition, (3) being bedridden or unable to walk without physical support of another person, (4) residence more than 40 km from the research center, (5) acute psychosis or social aggression, (6) inability to communicate verbally in German or English [24]. A threshold of 60 years was chosen, because heterogeneity concerning functional decline begins at an earlier age [25] underscoring the importance of initiating monitoring and prevention as early as possible.

The SeFallED study adheres to the Declaration of Helsinki. It has been prospectively registered in the German Clinical Trials Register (DRKS-ID: 00025949) and received approval from the Medical Ethics Committee of the University Oldenburg (number 2021 − 120). As part of the SeFallED study, participants were visited at home at around four weeks, six, twelve and 24 months after the initial fall that led to their ED presentation. During these home visits and after providing written consent, a comprehensive geriatric assessment was conducted, evaluating participants’ functionality, cognition, and wellbeing. The assessment was conducted as a personal interview. For this sub-study, data from the initial home assessment were used. Data collection was done within four weeks after presenting to the ED.

## Measures

### Socioeconomic status

In line with previous research, SES was measured using the two most common indicators for older adults: income and education [12]. Income was assessed via self-report using a single-item question on monthly net household income with predefined response categories. To calculate equivalent income, the reported household income was adjusted based on the number of household members using the modified equivalence scale of the Organization for Economic Co-operation and Development [26]. Accordingly, the income of a single-person household was divided by one, with each additional adult in the household adding a weight of 0.5. The question, answer categories, and explanation were based on the Socio-Economic Panel survey [27, 28] and the German Mikrozensus [29, 30], both of which are well-established German surveys. Participants chose from 24 income categories ranging from “under €250” to “€25,000 or more”. Furthermore, they had the option to not specify their income. To reduce potential discomfort in reporting income, participants entered their responses confidentially using a tablet. Income categories were subsequently converted into numerical values following previous research [31]. For statistical analyses, income was included as a continuous variable for the group comparisons and as a categorical variable for regression analyses. This approach was chosen to avoid data loss for regression analysis and to facilitate the calculation of linear regressions, which may be more interpretable than ordinal regressions. Following Lampert and colleagues [16], the categorical variable was created by dividing income into five groups based on the median of this sample. To categorize income, we used the following thresholds based on percentages of the median income of the sample: group 1: under 60%, group 2: 60% until below 80%, group 3: 80% until below 100%, group 4: 100% until below 150%, and group 5: 150% or more of the median income. In this study, the median income of the sample was used instead of that of the general population, as the sample exclusively comprises individuals aged 60 years and over, making the general population median unsuitable for this context. In Germany, income levels in older age are primarily determined by occupational history. Upon retirement, individuals receive a state pension, which is calculated based on lifetime earnings and the number of years spent in paid employment.

Education was assessed through the number of years in school. Based on the Comparative Analysis of Social Mobility in Industrial Nations classification [32], and in line with the German education system, participants were stratified into three groups: group 1 (less than ten school years), group 2 (equal to or more than ten, equal or less than twelve years), and group 3 (more than twelve years of scholastic education). The German education system offers different levels of school-leaving qualifications, which historically vary by federal state and over time. In this study, educational attainment was categorized as follows: Low education: completion of elementary school (Volksschule), low secondary education (“Hauptschule”) or no formal qualification; Medium education: completion of intermediate secondary school (e.g., Realschule); High education: qualification for university entrance (Abitur). This classification allows for a meaningful differentiation in terms of educational achievement.

### Health outcomes

Health outcomes were classified into four distinct groups and indicated: mental and functional performance, physical activity, mental well-being, or health care utilization. The health outcomes were selected from the SeFallED study to ensure representation across a broad spectrum of health domains, which are key indicators of long-term health and quality of life in older adults [33–36]. Health related quality of life, an indicator of well-being, was assessed with two tools: a questionnaire (EQ-5D-3L [37]) and a visual analogue scale (EQ-VAS [37]). The EQ-5D-3L covers five dimensions: mobility, self-care, usual activities, pain/discomfort and anxiety/depression [37]. It provides an index value that reflects health status based on population preferences [37]. In contrast, the EQ visual analogue scale (VAS) records an individual’s overall self-rated health on a visual analogue scale from 0 to 100, capturing a more subjective, holistic perception of well-being [37]. These differences in structure and perspective may lead to divergent results and are, therefore, complementary. Table 1 depicts the outcomes included in this analysis.

**Table 1:** Health outcomes and their operationalization

Outcome	Variable	Operationalization	Minimum and maximum of points
Mental and functional performance	Activities of Daily Living (ADLs)	Barthel-Index [38]	0-100 points, greater score indicates higher independence
	Cognitive function	Montreal Cognitive Assessment (MoCA) [39]	0–30 points, greater score indicates better cognitive function
	Hand grip strength	Hydraulic hand dynamometer, measured in kg, highest value from three measurements with dominant hand [40]	/
	Physical performance	Short Physical Performance Battery (SPPB) [41]	0–12 points, greater score indicates better physical functioning
Physical activity	Step count	Average steps per day, measured using activity monitor (activPAL© activity monitor; PAL Technologies Ltd., Glasgow, UK) worn on the thigh continuously for 5–7 days. Data were processed using PALconnect (PAL Technologies Ltd., Glasgow, UK) and PALanalysis software (PAL Technologies Ltd., Glasgow, UK) and PALanalysis (PAL Technologies Ltd., Glasgow, UK)	/
	Sedentary time	Average sedentary minutes per day, calculated using the activPAL© activity monitor (PAL Technologies Ltd., Glasgow, UK), worn on the thigh continuously for 5–7 days Data were processed using PALconnect (PAL Technologies Ltd., Glasgow, UK)and PALanalysis software (PAL Technologies Ltd., Glasgow, UK)	/
Mental wellbeing	Concerns about falling	German Short Falls Efficacy Scale (FES-I) [42, 43]	7–28 points, greater score indicates higher concerns
	Health-related quality of life (HrQoL) HrQoL – visual analogue scale (VAS)	EQ-5D-3L, questionnaire with dimensions: mobility, self-care, usual activities, pain/discomfort and anxiety/depression) [37], values of EQ-5D-3L were calculated following Greiner and colleagues [44] EQ visual analogue scale (VAS) measuring self-rated health [37]	EQ-5D-3L: −0.205–0.999 score, greater score indicates higher HrQoL; EQ-VAS: 0-100 points, greater value indicates higher HrQoL
Health care utilization	Follow-up physician visits	Follow-up physician visits after presentation to the emergency department up to four weeks after the initial fall, classified as “no utilization” and “utilization”	/
	Therapy appointments	Outpatient therapies, such as physical therapy or occupational therapy up to four weeks after the initial fall, classified as “no utilization” and “utilization”	/

### Covariates

Age, and sex were included as covariates due to their relevance as potential confounders and their established relationships with socioeconomic variables and health as shown in previous research [45, 46]. For instance, age and sex are associated with health outcomes such as concerns about falling [45]. In this sample, age may be associated with income, as older adults who are retired are likely to have lower incomes compared to younger older adults who are still employed. Similarly, income is associated with sex [46]; however, because household income was assessed, this association may be less pronounced. Nevertheless, controlling for the association of sex and income remains important, particularly as the analysis includes both multi-person and single-person households, where sex may play a more significant role. We based the selection of covariates on a minimal sufficient adjustment set to appropriately control for confounders and avoid bias. To ensure consistency and facilitate comparisons of associations across variables, a standardized set of covariates was used. Age was recorded in years, and sex was categorized as male or female.

### Statistical analysis

Normal distribution was evaluated with Kolmogorov-Smirnov tests. Given the non-normal distribution, variables were reported as median with interquartile range (25th and 75th percentiles). Categorical variables were summarized as frequencies. A *p*-value of < 0.05 was considered statistically significant. Group differences between participants who disclosed income and those who did not were analyzed using Mann-Whitney U tests (for age) and Chi-square tests (for sex and education).

Associations between SES indicators and health outcomes were analyzed using correlation analyses and regression models. For the main analyses, only participants who disclosed their monthly household income were included. Participants who did not report income were excluded from income-related analyses. To examine potential bias, group comparisons were conducted between individuals who reported income and those who did not.

Income and education were treated as categorical variables to facilitate group comparisons. Due to non-normally distributed variables, Kruskal-Wallis tests were applied. Post hoc pairwise comparisons with Bonferroni-adjusted *p*-values [47] were conducted to account for multiple testing. Differences in categorical outcomes were analyzed using Pearson chi-squared tests.

Spearman correlation and phi coefficients were calculated to assess the relationships between the variables included in this study. Cohen’s values of 0.1, 0.3, and 0.5 were considered small, medium and large effects, respectively [48].

Linear regression models were employed for numerical health outcomes, while binary logistic regression models were used for categorical health outcomes (follow-up physician visits and therapy appointments). Two regression models were calculated for each health outcome. The first model included equivalent household income and school years as independent, continuous variables. The second model contained age and sex as additional covariates to account for potential confounding. Multicollinearity was assessed using variance inflation factors (VIFs), with a threshold of five indicating multicollinearity [49]. Outliers were inspected if their studentized residuals were bigger than three [49]. In cases of non-normal residuals or suspected heteroscedasticity, bias-corrected and accelerated bootstrapping (1,000 samples, 95% confidence interval) was applied. A Mersenne Twister (seed 2,000,000), originally developed by Matsumoto and Nishimura [50], ensured reproducibility. Sensitivity analyses were conducted using ADLs as an example, assessing robustness against outliers, occupational status, age group variation and different combinations of covariates. Each regression model included participants with complete data on the respective dependent variable and the covariates included in the model. Therefore, the sample size varied across models depending on the availability of data. For binary logistic regression models, the Box-Tidwell-Method was used to test linearity [51], multicollinearity was inspected using a correlation matrix, and a model fit was assessed using Nagelkerkes R². All analyses were performed using SPSS Statistics (Version 28, Armonk, NY: IBM Corp). The manuscript follows the STROBE reporting guidelines [52].

## Results

### Sample characteristics

The SeFallED study enrolled 335 participants in total. Due to participant dropouts and the later introduction of income assessment, not all 335 participants could be approached for this sub-study. We addressed 204 participants to attain income data, of these, 32 (15.7%) did not disclose their income resulting in a final sample size of 172 participants. The participants’ ages ranged from 60 to 93 years, with a median age of 74 and an interquartile range from 66 to 81 years. The median income was €2,125, €1,416 in 25th percentile and €2,833 in 75th percentile. Participants attended school for a median of 10 years with an interquartile range of 9 to 12 years (25th to 75th percentile). The characteristics of the participants are summarized in Table 2.

**Table 2:** Participants baseline demographics and clinical characteristics

Baseline demographics						
	**n**	**%**				
Total number of participants	172					
Sex						
Male/Female	72/100	41.9%/58.1%				
Income						
< €1275	30	17.4%				
≥ €1275 < €1700	25	14.5%				
≥ €1700 < €2125	29	16.9%				
≥ €2125 < €3178	73	42.4%				
≥ €3178	15	8.7%				
Education						
< 10 years in school	68	39.5%				
≥ 10 ≤ 12 years in school	74	43.0%				
> 12 years in school	30	17.4%				
Occupational status						
Working/Retired	32/140	18.6%/81.4%				
Household size						
One-person household	70	40.7%				
Two-person household	100	58.1%				
More than two-person household	2	1.2%				
**Clinical characteristics**						
	**n**	**median [25th percentile**, **75th percentile]**			**min**	**max**
Mental and functional performance						
ADLs	170	100 [95.00,100.00]			35	100
Cognitive status (MoCA)	170	24 [21.00,27.00]			11	30
Hand grip strength	154	29 [22.00, 38.25]			1	60
Physical performance (SPPB)	154	10 [8.00, 11.00]			1	12
Physical activity						
Step count	128	5858 [3840.50, 8571.00]			11	19,220
Sedentary time	127	662.29 [537.63, 737.35]			343	1054
Mental well-being						
Concerns about falling (FES-I)	169	9 [7.00, 11.00]			7	28
Health-related quality of life (HrQoL, EQ-5D-3L)	172	0.88 [0.78, 0.99]			−0.1	0.9
HrQoL– visual analogue scale (HrQoL VAS, EQ VAS)	171	70 [50.00, 80.00]			5	100
Health care utilization						
Follow-up physician visits (yes)/(no)	79/93	-			-	-
Therapy appointments (yes)/(no)	114/58	-			-	-

*SD* standard deviation, Activities of Daily Living (ADLs):0-100 points, cognitive status: 0–30 points, *MoCA* Montreal Cognitive Assessment, physical performance: 0–12 points, *SPPB* Short Physical Performance Battery, concerns about falling: 7–28 points, *FES-I* German Short Falls Efficacy Scale International, Health-related quality of life (HrQoL):−0.205−0.999 points, Health-related quality of life visual analogue scale (HrQoL-VAS):0-100%

Participants who did not disclose their income, had an average age of 73.7 ± 9.6 years, were predominantly female (6 males/26 females) and had attended school for approximately 10.1 ± 2,19 years. Compared to the income-disclosing participants, these differences were not statistically significant for age (U = 2691.00, z = − 0.0199, *p* =.842) and education (U = 2545.00, z = −0.683, *p* =.842), but were significant for sex (χ²(1, *n* = 204) = 6.102, *p* =.014) with a higher proportion of females in the group that did not disclose their income compared to the income-disclosing participants.

### Group comparison

The results of the group comparisons for health outcomes across education groups are presented in Table 3 with detailed results provided in Supplementary Material– Table S1. Significant effects were observed for cognitive status across income groups (H(4) = 10.392, *p* =.034); however, this effect did not remain significant after Bonferroni correction. For mental and functional performance, significant differences remained after Bonferroni correction. Individuals in the group with less than ten years in school had worse ADLs than individuals with ten to twelve years in school (*p* =.005) and individuals with more than twelve years of school (*p* =.009). Furthermore, individuals with the lowest number of years in school had a lower cognitive status than individuals with more than twelve years of school (p = < 0.001) and a lower hand grip strength compared to both individuals with ten to twelve school years (*p* =.001) and with more than twelve school years (p = < 0.001). Physical performance was also lower in individuals with less than 10 years of school in comparison to individuals with more than twelve years of school (*p* =.002). Groups stratified by income or education did not differ significantly in regard to physical activity or mental well-being. Chi-square tests indicated significant differences for education groups in relation to follow-up physician visits (χ²(2) = 6.638, *p* =.036), with higher education leading to more physician visits.

**Table 3:** Group comparisons for health outcomes across education groups

	Test Statistics (H)	*p*-value	Post hoc comparison (group 1: < 10 years in school, group 2: ≥ 10 ≤ 12 years in school, group 3: > 12 years in school)		
			**1–2**	**1–3**	**2–3**
Mental and functional performance					
Activities of daily living (ADLs)	13.469	0.001**	0.005**	0.009**	1.000
Cognitive status (MoCA)	18.391	< 0.001**	0.084	< 0.001**	0.027*
Hand grip strength	20.614	< 0.001**	0.001**	< 0.001**	0.735
Physical performance (SPPB)	12.199	0.002**	0.082	0.002**	0.269
Physical activity					
Step count	1.036	0.596	1.000	0.949	1.000
Sedentary time	2.280	0.320	0.516	0.725	1.000
Mental well-being					
Concerns about falling (FES-I)	2.326	0.313	0.923	0.441	1.000
Health-related quality of life (HrQoL, EQ-5D-3L)	1.663	0.435	1.000	0.652	0.769
HrQoL – visual analogue scale (VAS, EQ VAS)	0.022	0.989	1.000	1.000	1.000
	**Test statistics (χ²**)	**p-value**	**Post hoc comparison 1–2**	**Post hoc comparison 1–3**	**Post hoc comparison 2–3**
Health care utilization					
Follow-up physician visits	6.638	0.036*	1.000	0.118	0.032*
Therapy appointments	0.922	0.631	1.000	1.000	1.000

*ADLs *0-100 points, cognitive status: 0–30 points, *MoCA* Montreal Cognitive Assessment, physical performance: 0–12 points, *SPPB* Short Physical Performance Battery, concerns about falling: 7–28 points, *FES-I* German Short Falls Efficacy Scale, HrQoL: −0.205−0.999 points, HrQoL-VAS:0-100%, * *p* <.05. ** *p* <.01

Significant age differences were observed across education groups (H(2) = 21.418, *p* =.001), with individuals in the lowest education group being older on average. No significant age differences were found across income groups (H(4) = 0.595, *p* = 964). The chi-square test indicated significant differences in income between sexes (χ²(4) = 10.463, *p* =.033) with a tendency of women having lower incomes. However, no significant differences were found for education between sexes (χ²(2) = 1.238, *p* =.539).

### Correlation analysis

Positive associations were identified between education and various health outcomes. Correlation analysis revealed small to medium positive associations for ADLs (*r* =.297, *p* <.001), cognitive status (*r* =.331, *p* <.001), hand grip strength (*r* =.356, *p* <.001), as well as physical performance (*r* =.276, *p* <.001). Similarly, positive associations were observed between income and ADLs (*r* =.172, *p* =.025), cognitive status (*r* =.200, *p* =.009), and physical performance (*r* =.285, *p* <.001). Conversely, a negative association was found between income and concerns about falling (*r* = −.180, *p* =.020). No further associations between the socioeconomic status and health outcomes were found. See Supplementary Material- Table S2 for the full results.

### Regression analysis

VIFs were below 1.5 for all regression models. Detailed results of the regression analysis for Model 1 are provided in Supplementary Material- Table S3. Model 1, including education and income as independent variables, showed statistically significant associations for ADLs (*p* <.001), cognitive status (*p* <.001), hand grip strength (*p* <.001), and physical performance (*p* <.004). Individuals with a higher education had better mental and functional outcomes, whereas income was not associated with any of these outcomes. Model 1 revealed no further associations of education or income with step count (*p* =.740), sedentary time (*p* =.277) and concerns about falling (*p* =.276), HrQoL (*p* =.641) as well as with the HrQoL visual analogue scale (VAS, *p* =.919). Logistic regression models for health care utilization (follow-up physician visits (*p* =.158) and therapy appointments (*p* =.512)) did not yield statistically significant results for income and education.

The results of the regression analysis for Model 2 are presented in Table 4. In Model 2, age and sex were added as variables. Model 2 revealed statistically significant differences for ADLs (*p* <.001), cognitive status (*p* <.001), hand grip strength (*p* <.001), physical performance (*p* <.001)), step count (*p* =.004), sedentary time (*p* =.014) and HrQoL (*p* =.041). However, the regression analysis for concerns about falling (*p* =.059), HrQoL VAS (*p* =.123), follow-up physician visits (*p* =.075) as well as therapy appointments (*p* =.098) remained non-significant. Similar to Model 1, individuals with higher education exhibited better mental and functional performance. Furthermore, income remained insignificant. Covariates were also associated with health outcomes, as older adults had lower mental and functional performance, had a lower number of steps per day and a slightly lower quality of life. Females had a higher cognitive status, lower hand grip strength and were less sedentary compared to males. Results from additional sensitivity analyses, including models with and without outliers, with and without retirees, for different age groups, and for different combinations of covariates can be found in Supplementary Tables S4- S7.

**Table 4:** Results of regression analysis for health outcomes

		bootstrapped B-value	95% confidence interval	bootstrapped standard error	*p*-value
Mental and functional performance					
Activities of daily living (ADLs) ^a^	Intercept	114.345	[102.104, 127.768]	7.213	< 0.001**
Overall *p*-value = < 0.001, adjusted R²=0.174	Income	0.000	[0.000, 0.003]	0.001	0.518
	Education	1.029	[0.311, 1.719]	0.440	0.028*
	Age	−0.381	[−0.583, −0.179]	0.096	0.003**
	Sex	−1.144	[−3.947, 1.743]	1.484	0.460
Cognitive status (MoCA) ^b^	Intercept	29.361	[23.081, 35.861]	3.087	< 0.001**
Overall *p*-value = < 0.001, adjusted R²=0.294	Income	0.000	[0.000, 0.001]	0.000	0.322
	Education	0.480	[0.184, 0.714]	0.162	0.005**
	Age	−0.173	[−0.239, −0.119]	0.032	< 0.001**
	Sex	1.145	[0.194, 2.145]	0.479	0.018*
Hand grip strength ^c^	Intercept	87.615	[74.713, 101.679]	7.252	< 0.001**
Overall *p*-value = < 0.001, adjusted R²=0.604	Income	0.000	[−0.001, 0.002]	0.001	0.244
	Education	1.056	[0.350, 1.649]	0.355	0.007**
	Age	−0.598	[−0.746, −0.466]	0.072	< 0.001**
	Sex	−14.693	[−17.367, −11.750]	1.342	< 0.001**
Physical performance (SPPB)^d^	Intercept	15.073	[11.864, 18.052]	1.640	< 0.001**
Overall *p*-value = < 0.001, adjusted R²=0.195	Income	0.000	[0.000, 0.001]	0.000	0.657
	Education	0.184	[−0.073, 0.385]	0.116	0.121
	Age	−0.112	[−0.154, −0.076]	0.021	< 0.001**
	Sex	0.232	[−0.457, 0.991]	0.364	0.539
Physical activity					
Step count^e^	Intercept	16730.219	[9299.122, 24413.882]	4300.472	< 0.001**
Overall *p*-value = 0.004, adjusted R²=0.087	Income	0.087	[−0.375, 1.058]	0.281	0.416
	Education	−111.164	[−499.147, 258.869]	198.201	0.580
	Age	−139.460	[−224.045, −60.291]	37.500	< 0.001**
	Sex	632.310	[−745.242, 1878.1816]	630.842	0.326
Sedentary time ^f^	Intercept	483.796	[144.479, 794.572]	173.337	0.007**
Overall *p*-value = 0.014, adjusted R²=0.067	Income	−0.001	[−0.014, 0.041]	0.012	0.895
	Education	11.449	[−1.660, 24.656]	7.046	0.105
	Age	2.245	[−0.574, 5.097]	1.554	0.150
	Sex	−71.428	[−131.622, −11.539]	26.499	0.007**
Mental well-being					
Concerns about falling (FES-I)^g^	Intercept	3.461	[−2.840, 9.931]	3.208	0.283
Overall *p*-value = 0.059, adjusted R²=0.030	Income	0.000	[−0.001, 0.000]	0.000	0.746
	Education	−0.121	[−0.471, 0.340]	0.168	0.456
	Age	0.095	[0.023, 0.167]	0.037	0.008**
	Sex	0.569	[−0.829, 1.875]	0.660	0.394
Health-related quality of life (HrQoL. EQ-5D-3 L) ^h^	Intercept	1.314	[0.898, 1.713]	0.203	< 0.001**
Overall *p*-value = 0.041, adjusted R²=0.035	Income	0.000	[0.000, 0.000]	0.000	0.538
	Education	−0.003	[−0.021, 0.015]	0.010	0.752
	Age	−0.006	[−0.010, −0.002]	0.002	0.011*
	Sex	−0.025	[−0.091, 0.048]	0.034	0.481
HrQoL – visual analogue scale (VAS, EQ VAS) ^i^	Intercept	109.398	[71.083, 147.746]	19.360	< 0.001**
Overall *p*-value = 0.123, adjusted R²=0.019	Income	0.000	[−0.001, 0.006]	0.002	0.855
	Education	−1.019	[−2.656, 0.328]	0.945	0.281
	Age	−0.499	[−0.901, −0.109]	0.202	0.014*
	Sex	2.282	[−0.109, 8.917]	3.291	0.509
			**95% confidence interval**	**odds ratio**	***p*** **-value**
Health care utilization					
Follow- up physician visit ^j^	Intercept			0.037	0.084
Overall *p*-value = 0.075, adjusted R²=0.064	Income		[1.000,1.000]	1.000	0.304
	Education		[0.943, 1.349]	1.127	0.189
	Age		[0.979, 1.053]	1.016	0.408
	Sex		[1.029, 3.648]	1.937	0.041*
Therapy appointments ^k^	Intercept			0.640	0.822
Overall *p*-value = 0.098, adjusted R²=0.062	Income		[1.000, 1.001]	1.000	0.331
	Education		[0.813, 1.168]	0.983	0.857
	Age		[0.969, 1.046]	1.006	0.753
	Sex		[1.205, 4.449]	2.316	0.012*

*Sex* reference group are male adults, *ADLs* 0-100 points, cognitive status: 0–30 points, *MoCA* Montreal Cognitive Assessment, physical performance: 0–12 points, *SPPB* Short Physical Performance Battery, concerns about falling: 7–28 points, *FES-I* Germany Short Falls Efficacy Scale, *HrQoL* −0.205−0.999 points, *HrQoL-VAS* = 0-100%

^a^*n* = 169 for Model 2, ^b^*n* = 169 for Model 2, ^c^*n* = 153 for Mode*l 2,*^d^*n* = 153 for Model 2, ^e^*n* = 127 for Model 2, ^f^*n* = 126 for Model 2, ^g^*n* = 168 for Model 2, ^h^*n* = 171 for Model 2, ^i^*n* = 170 for Model 2, ^j^*n* = 172 for Model 2, ^k^*n *= 172 for Model 2, *p <.05. **p <.001

## Discussion

### Main results

The present study analyzed associations between income, education and various health outcomes in older adults who experienced an acute severe fall. Adjusted R² values indicated that the regression model including age and sex provided a better fit. The findings demonstrated that education had the strongest associations with health outcomes, particularly with mental and functional performance. Income had little to no associations with health outcomes but was negatively correlated with concerns about falling, indicating that individuals with higher income had fewer concerns about falling.

The associations observed in the present study are consistent with previous literature; however, earlier studies have reported stronger associations with both income and education. For example, associations between ADLs and both education and income were identified in adults aged 45 years and older in China [53], while hand grip strength was found to be associated with education, particularly in individuals over 50 [54]. Additionally, physical functioning was linked to both income and education in adults aged 55 or older [55], and low education and income were identified as risk factors for cognitive impairment and dementia [56]. In contrast to our findings, previous studies have reported associations between income, education and concerns about falling in participants aged 65 and older [57], as well as between physical activity and income in those over 60 [58] and quality of life with both education and income in individuals over 60 [59]. Furthermore, higher SES has been associated with more frequent use of physical therapy services in adults [60]. The limited associations with income or education and health outcomes observed in this study may be attributed to several factors. One of the most notable is the specificity of the study population, which consists of older adults who experienced a severe fall and were recruited from the ED. In contrast, many previous studies include broader populations, often with younger participants or individuals from different clinical or community settings. For instance, older adults with low income who survive into later life may represent a particularly healthy subgroup within their income bracket [61], potentially reducing the observable differences in our sample. However, there is no information about the income groups of persons who declined participation. The primary reason for non-participation in the SeFallED study was reported to be feeling too unwell [62], which may lend support to the aforementioned speculation that healthier individuals with low income are more likely to participate in the study.

Additionally, the impact of education may be influenced by generational effects. In this study, age varied across education groups, which is consistent with data from the Mikrozensus [63]. Individuals aged over 75 were more likely to have completed elementary school (“Volksschulabschluss”) whereas younger individuals (aged 55 to 65) more frequently attained a high school diploma (“Abitur”) [63]. Educational systems and standards have evolved significantly over time and differ between countries, which may affect both how education is categorized and how strongly it relates to health outcomes. Therefore, differences in age distributions, cultural and educational contexts, and study settings likely contribute to the variation in findings across studies.

Additionally, individuals who were hospitalized after the fall were excluded, which may have influenced the sample, as some research suggests that fall severity may vary by SES. While some studies found no SES differences in fall-related deaths [64], others reported higher post-fall mortality in men with low education [20]. Besides the fall being more severe, it can also be speculated that individuals with lower SES may be more frequently admitted to inpatient care, as their living arrangements or social network may be less equipped to provide immediate follow-up care after an ED visit [65]. Including individuals with inpatient treatment after a fall in future studies could provide further insights into the associations between severe falls and SES. Moreover, longitudinal analyses in future research could help determine whether associations between SES and health outcomes evolve over time. This would offer valuable information for the implementation of follow-up care strategies, tailored interventions as advocated by the World Falls Guidelines [7], and highlight the importance of considering SES in treatment planning.

Differences between the present study’s findings and those in literature may also stem from variations in assessing SES. Previous studies have measured education using similar metrics such as the number of school years [9], but also through the highest qualification [54] or overall years of education, including vocational training and college [55]. Similarly, income has been assessed in various ways, including self-evaluation of economic status [53], annual household income [54] or monthly net income [55]. These approaches may capture different aspects of financial status, and each comes with its own strengths and limitations. As SES is not a uniformly defined construct, the use of standardized, validated indicators would enhance comparability across studies. Future research should, therefore, prioritize consensus on the operationalization of SES in older populations.

It is essential to recognize that different measurement approaches may influence the observed associations. In this study, associations between HRQoL and socioeconomic parameters were identified, but not for the HrQoL VAS. This discrepancy could be due to different focuses of these assessments, as the VAS may capture health on a broader scale compared to the overall HRQoL score obtained from the EQ-5D-3L questionnaire [66]. Additionally, research suggests that the direction of associations between physical activity and SES may vary depending on whether physical activity is measured using sensor data or self-reports [67]. Self-reports are often prone to inaccuracies, with participants often overreporting their activity levels [68]. In contrast, physical activity measured through activity sensors, which was done in this study, may provide a more accurate estimate. Thus, exploring various measurement methods and their associations with SES could provide valuable insights for future research. In this context, it is important to note that the sensor was worn voluntary, resulting in a reduced sample size for sedentary time and step count. This may have introduced selection bias, but the use of device-based measurement was prioritized due to its superior accuracy compared to self-report.

Around 16% of the approached individuals did not disclose their income, which may have introduced selection bias into our analyses [69], because this sample was excluded for main analyses. Previous studies suggest that individuals who refrain from reporting their household income often have more complex financial situations [70], and are more likely to belong to the lowest or highest income deciles [70]. Additionally, non-disclosure is more common among women [12, 70], which is consistent with our findings. In our sample, women were more likely to choose not to report income. These patterns underline the need to improve financial literacy and transparency across all age groups, particularly among women, to enable a more accurate assessment of socioeconomic inequalities in health. Future studies should investigate reasons for income non-disclosure in more detail to better understand and address potential sources of bias. The statistically relevant discrepancy may be partly explained by factors such as education level, marital status, age, and traditional societal roles of women [71]. Therefore, enhancing financial literacy across age, particularly among women, may be crucial.

In our study, education was associated with mental and functional performance, although the strength of these associations weakened when age and sex were controlled for in the regression models. Sensitivity analyses for ADLs showed that education explained an additional 3.1% of the variance beyond age. Interestingly, adding sex to the model slightly reduced the model fit. These findings underscore the importance of including age as a key covariate when analyzing associations between SES and health outcomes. While the proportion of variance explained by education is modest, the primary focus of this study is to investigate the association of SES and health, justifying the inclusion of education in our models.

While previous studies confirm the associations between education and health outcomes, they also emphasize the importance of mediating factors. One such factor is health literacy, which has been shown to be relevantly associated with both educational attainment and health outcomes in adults [72], including older adults. These findings highlight the importance of promoting health literacy across the lifespan, particularly in populations with lower educational levels. Strengthening health literacy may represent a key strategy to mitigate health disparities. A higher health literacy may be beneficial for reducing fall risk in aging populations, which could thereby increase the relevance of findings for public health.

### Strengths and limitations

Recruiting participants in EDs offers the opportunity to include a more diverse group in research, as EDs are frequently visited by individuals with lower SES and those from rural areas [73], populations that are often underrepresented in healthcare research. This recruitment strategy provides valuable insights into the relationship between SES and health outcomes due to the heterogeneity of the study population, resulting in a more representative sample [73]. This analysis specifically focused on older adults who experienced a severe fall, a group that is often underrepresented in research despite falls being one of the leading causes of ED visits among older adults [74]. This highlights the importance of studying the associations between SES and health outcomes in this population, as it is a significant clinical concern. Another strength of the study is its examination of a broad range of health outcomes. By separately analyzing the contribution of income and education, the study allows for more specific conclusions, which can inform future research directions. The broad heterogeneity of the sample is also reflected by the distribution of income. Overall, the median income of our sample was slightly higher than the income of adults in the region in Northern Germany (“Weser-Ems”) where we recruited participants [75] and also higher than national averages for adults aged 65 and older in Germany [76]. The slightly elevated median income may, in part, be attributed to non-response bias - specifically, the underrepresentation of individuals with lower incomes who were less likely to disclose their financial information, as observed in previous research [70].“.

One limitation of the study is that both income and education were self-reported, which may introduce bias through misreporting or non-response. Research suggests that inaccuracies in reporting may depend on socioeconomic characteristics, such as education level or income itself [77]. To help mitigate this, we included more than one SES parameter (education and income) in our study. While income data were collected using a digital tablet with detailed instructions to enhance understanding and privacy, approximately 16% of participants chose not to report their income. The non-response rate is similar to that observed in other large-scale surveys, such as the German Socioeconomic Panel Study [70]. Another limitation is that income was categorized rather than assessed as an absolute value, which may lack precision [78]. Nevertheless, this approach likely reduced participant burden, allowing for quicker assessments. The number of school years may be influenced by political and societal circumstances, leading to homogeneity within age groups [12]. There is also the potential for selection bias, as participants willing to engage in research may generally have a higher level of education [79], a trend evident in recruitment numbers [62]. Long-term follow-up is needed to further investigate the future consequences of differences in education or income.

While income and education are the most commonly used indicators of the socioeconomic status in older adults, additional measures – such as the satisfaction with income, former occupational status, or wealth – could provide a more comprehensive understanding. However, the primary aim of the SeFallED study was not to exhaustively assess SES, but to explore trajectories of functional decline following a fall. Future research may benefit from incorporating a broader range of socioeconomic indicators to deepen the analysis of associations observed with mental and functional performance.

Participants were recruited from a specific region in Germany, which may introduce selection bias. However, adults living in regions in and around Oldenburg experience significant income inequalities, as measured by taxes [80]. To better capture SES beyond the personal level (i.e. income and education), future research should aim to incorporate neighborhood- or community-level factors, such as multiple and social deprivation. While this was not feasible in our study due to limited data availability, such measures are especially relevant in ED-based samples, which tend to be diverse. Although we recruited from a defined region our participants resided within a 40 km radius of the study center, encompassing both urban areas with high population density of 1,652.82 inhabitants/km² (City of Oldenburg) and rural regions (e.g. 79.11 inhabitants/km² in Elsfleth) [81].

## Conclusion

This study examined the associations between SES and health outcomes in older adults following an acute severe fall. Significant associations were identified between education and mental and functional performance in both group comparisons and regression analyses, as well as correlations between income, education, and health outcomes related to mental and functional performance. These results provide insights into the associations between socioeconomic status and health outcomes within this specific population and may inform future research aimed at improving mental and functional performance through targeted prevention strategies. Such prevention programs could benefit from presenting study information in plain language and recruiting participants from socioeconomically diverse settings to reach those most likely to benefit. Furthermore, the involvement of a patient and stakeholder board appears crucial for reducing selection bias and addressing individuals at highest risk for functional decline. This approach also acknowledges the heterogeneity of clinical care in the ED, which should be emphasized in training ED staff.

## Supplementary Information

Supplementary Material 1.

## Data Availability

The datasets used and/or analyzed during the current study are available from the corresponding author on reasonable request.

## Abbreviations

ADLs: Activities of Daily Living
ED: Emergency Department
FES-I: Falls Efficacy Scale -International
HRQoL: Health-Related Quality of Life
MoCA: Montreal Cognitive Assessment
SD: Standard Deviation
SeFallED: Sentinel Fall Presenting to the Emergency Department
SES: Socioeconomic Status
SPPB: Short Physical Performance Battery
VAS: Visual Analogue Scale
VIFs: Variance Inflation Factors
