# Supplementary material for: How socioeconomic status shapes health outcomes following severe falls: a cross-sectional analysis
Source: BMC Geriatr. 2025 Jul 16;25:533. doi: 10.1186/s12877-025-06198-9 (PMC12269145; doi:10.1186/s12877-025-06198-9)
Supplement: Supplementary file 1 — Supplementary Material 1. [file 12877_2025_6198_MOESM1_ESM.docx]

# **Supplementary Material for “****How** **Socioeconomic Status Shapes Health Outcomes Following Severe Falls: A Cross-Sectional Analysis”**

Supplementary Material for “How Socioeconomic Status Shapes Health Outcomes Following Severe Falls: A Cross-Sectional Analysis”

by Elisa-Marie Speckmann, Lars Schwettmann, Laura Himmelmann, Tania Zieschang, Tim Stuckenschneider in the Journal of Geriatrics

**Table S1: Group comparisons stratified by income and education for health outcomes.**

| Variable | |  | Test Statistics (H) | Degrees of freedom | p-value | n | Mean rank | Pairwise Comparison | Test Statistics (H) | Standard deviation | | Adjusted signi-ficance | |  |
| --- | --- | --- | --- | --- | --- | --- | --- | --- | --- | --- | --- | --- | --- | --- |
| Mental and functional performance |  |  | |  |  |  |  |  |  | |  | |  |  |
| Activities of Daily Living (ADLs, n=170) | | Income | 6.053 | 4 | .195 | 1=30 | 1=71.70 | 1-2 | -16.140 | | 10.497 | | 1.000 |  |
|  | |  |  |  |  | 2=25 | 2=87.84 | 1-3 | -11.472 | | 10.094 | | .256 |  |
|  | |  |  |  |  | 3=29 | 3=83.17 | 1-4 | -17.210 | | 8.423 | | .410 |  |
|  | |  |  |  |  | 4=72 | 4=88.91 | 1-5 | -26.479 | | 12.546 | | .348 |  |
|  | |  |  |  |  | 5=14 | 5=98.18 | 2-3 | -4.668 | | 10.578 | | 1.000 |  |
|  | |  |  |  |  |  |  | 2-4 | -1.070 | | 8.998 | | 1.000 |  |
|  | |  |  |  |  |  |  | 2-5 | -10.339 | | 12.939 | | 1.000 |  |
|  | |  |  |  |  |  |  | 3-4 | -5.737 | | 8.525 | | 1.000 |  |
|  | |  |  |  |  |  |  | 3-5 | -15.006 | | 12.614 | | 1.000 |  |
|  | |  |  |  |  |  |  | 4-5 | -9.269 | | 11.322 | | 1.000 |  |
|  | | Education | 13.469 | 2 | .**001**** | 1=68 | 1=72.33 | 1-2 | -20.480 | | 6.511 | | **.005**** |  |
|  | |  |  |  |  | 2=74 | 2=92.81 | 1-3 | -25.830 | | 8.704 | | **.009**** |  |
|  | |  |  |  |  | 3=28 | 3=98.16 | 2-3 | -5.350 | | 8.600 | | 1.000 |  |
| Cognitive status (n=170) | | Income | 10.392 | 4 | **.034*** | 1=30 | 1=72.57 | 1-2 | -25.350 | | 13.417 | | .588 |  |
|  | |  |  |  |  | 2=24 | 2=97.92 | 1-3 | -2.594 | | 12.873 | | 1.000 |  |
|  | |  |  |  |  | 3=28 | 3=75.16 | 1-4 | -12.043 | | 10.624 | | 1.000 |  |
|  | |  |  |  |  | 4=73 | 4=84.61 | 1-5 | -42.567 | | 15.492 | | .060 |  |
|  | |  |  |  |  | 5=15 | 5=115.13 | 2-3 | -22.756 | | 13.628 | | .950 |  |
|  | |  |  |  |  |  |  | 2-4 | -13.307 | | 11.527 | | 1.000 |  |
|  | |  |  |  |  |  |  | 2-5 | -17.217 | | 16.125 | | 1.000 |  |
|  | |  |  |  |  |  |  | 3-4 | -9.499 | | 10.890 | | 1.000 |  |
|  | |  |  |  |  |  |  | 3-5 | -39.973 | | 15.675 | | .108 |  |
|  | |  |  |  |  |  |  | 4-5 | -30.524 | | 13.888 | | .280 |  |
|  | | Education | 18.391 | 2 | **<.001**** | 1=66 | 1=69.45 | 1-2 | -18.241 | | 8.294 | | .084 |  |
|  | |  |  |  |  | 2=74 | 2=87.7 | 1-3 | -45.929 | | 10.787 | | **.000**** |  |
|  | |  |  |  |  | 3=30 | 3=115.38 | 2-3 | -27.687 | | 10.604 | | **.027*** |  |
| Hand grip strength (n=154) | | Income | 3.877 | 4 | .423 | 1=28 | 1=67.39 | 1-2 | -7.039 | | 12.696 | | 1.000 |  |
|  | |  |  |  |  | 2=22 | 2=74.43 | 1-3 | -11.570 | | 12.020 | | 1.000 |  |
|  | |  |  |  |  | 3=27 | 3=78.96 | 1-4 | -11.052 | | 10.121 | | 1.000 |  |
|  | |  |  |  |  | 4=63 | 4=78.44 | 1-5 | -28.071 | | 14.586 | | .543 |  |
|  | |  |  |  |  | 5=14 | 5=95.46 | 2-3 | -4.531 | | 12.799 | | 1.000 |  |
|  | |  |  |  |  |  |  | 2-4 | -4.013 | | 11.036 | | 1.000 |  |
|  | |  |  |  |  |  |  | 2-5 | -21.032 | | 15.325 | | 1.000 |  |
|  | |  |  |  |  |  |  | 3-4 | -0.519 | | 10.250 | | 1.000 |  |
|  | |  |  |  |  |  |  | 3-5 | -16.501 | | 14.676 | | 1.000 |  |
|  | |  |  |  |  |  |  | 4-5 | -17.020 | | 13.167 | | 1.000 |  |
|  | | Education | 20.614 | 2 | **<.001**** | 1=58 | 1=57.22 | 1-2 | -29.218 | | 7.938 | | **.001**** |  |
|  | |  |  |  |  | 2=69 | 2=86.44 | 1-3 | -40.980 | | 10.382 | | **.000**** |  |
|  | |  |  |  |  | 3=27 | 3=98.20 | 2-3 | -11.762 | | 10.116 | | .735 |  |
| Physical performance (n=154) | | Income | 5.982 | 4 | .201 | 1=26 | 1=66.27 | 1-2 | -0.936 | | 12.921 | | 1.000 |  |
|  | |  |  |  |  | 2=21 | 2=65.33 | 1-3 | -12.027 | | 12.101 | | 1.000 |  |
|  | |  |  |  |  | 3=27 | 3=78.30 | 1-4 | -15.753 | | 10.175 | | 1.000 |  |
|  | |  |  |  |  | 4=67 | 4=82.02 | 1-5 | -28.385 | | 14.959 | | .578 |  |
|  | |  |  |  |  | 5=13 | 5=94.65 | 2-3 | -12.963 | | 12.813 | | 1.000 |  |
|  | |  |  |  |  |  |  | 2-4 | -16.689 | | 11.014 | | 1.000 |  |
|  | |  |  |  |  |  |  | 2-5 | -29.321 | | 15.541 | | .592 |  |
|  | |  |  |  |  |  |  | 3-4 | -3.726 | | 10.039 | | 1.000 |  |
|  | |  |  |  |  |  |  | 3-5 | -16.358 | | 14.867 | | 1.000 |  |
|  | |  |  |  |  |  |  | 4-5 | -12.631 | | 13.347 | | 1.000 |  |
|  | | Education | 12.199 | 2 | **.002**** | 1=58 | 1=63.62 | 1-2 | -17.372 | | 7.871 | | .082 |  |
|  | |  |  |  |  | 2=68 | 2=80.99 | 1-3 | -34.147 | | 10.134 | | **.002**** |  |
|  | |  |  |  |  | 3=28 | 3=97.77 | 2-3 | -16.775 | | 9.889 | | .269 |  |
| Physical activity | |  |  |  |  |  |  |  |  | |  | |  |  |
| Step count (n=128) | | Income | 2.301 | 4 | .681 | 1=20 | 1=70.2 | 1-2 | -11.095 | | 11.884 | | 1.000 |  |
|  | |  |  |  |  | 2=19 | 2=59.11 | 1-3 | -14.512 | | 11.590 | | 1.000 |  |
|  | |  |  |  |  | 3=21 | 3=56.05 | 1-4 | -3.709 | | 9.686 | | 1.000 |  |
|  | |  |  |  |  | 4=55 | 4=66.49 | 1-5 | -1.354 | | 13.215 | | 1.000 |  |
|  | |  |  |  |  | 5=13 | 5=68.85 | 2-3 | -3.058 | | 11.745 | | 1.000 |  |
|  | |  |  |  |  |  |  | 2-4 | -7.386 | | 9.871 | | 1.000 |  |
|  | |  |  |  |  |  |  | 2-5 | -9.741 | | 13.352 | | 1.000 |  |
|  | |  |  |  |  |  |  | 3-4 | -10.443 | | 9.515 | | 1.000 |  |
|  | |  |  |  |  |  |  | 3-5 | -12.799 | | 13.091 | | 1.000 |  |
|  | |  |  |  |  |  |  | 4-5 | -2.355 | | 11.440 | | 1.000 |  |
|  | | Education | 1.036 | 2 | .596 | 1=47 | 1=61.82 | 1-2 | -2.020 | | 7.338 | | 1.000 |  |
|  | |  |  |  |  | 2=56 | 2=63.84 | 1-3 | -9.291 | | 9.182 | | .949 |  |
|  | |  |  |  |  | 3=25 | 3=71.02 | 2-3 | -7.181 | | 8.922 | | 1.000 |  |
| Sedentary time (n=127) | | Income | 3.038 | 4 | .552 | 1=20 | 1=60.40 | 1-2 | -4.956 | | 11.958 | | 1.000 |  |
|  | |  |  |  |  | 2=18 | 2=55.44 | 1-3 | -2.981 | | 11.500 | | 1.000 |  |
|  | |  |  |  |  | 3=21 | 3=63.38 | 1-4 | -4.691 | | 9.611 | | 1.000 |  |
|  | |  |  |  |  | 4=55 | 4=65.09 | 1-5 | -17.369 | | 13.113 | | 1.000 |  |
|  | |  |  |  |  | 5=13 | 5=77.77 | 2-3 | -7.937 | | 11.822 | | 1.000 |  |
|  | |  |  |  |  |  |  | 2-4 | -9.646 | | 9.994 | | 1.000 |  |
|  | |  |  |  |  |  |  | 2-5 | -22.325 | | 13.396 | | .956 |  |
|  | |  |  |  |  |  |  | 3-4 | -1.710 | | 9.441 | | 1.000 |  |
|  | |  |  |  |  |  |  | 3-5 | -14.388 | | 12.989 | | 1.000 |  |
|  | |  |  |  |  |  |  | 4-5 | -12.678 | | 11.351 | | 1.000 |  |
|  | | Education | 2.280 | 2 | .320 | 1=47 | 1=57.57 | \| 1-2 \| \| --- \| | -9.989 | | 7.311 | | .516 |  |
|  | |  |  |  |  | 2=55 | 2=67.56 | 1-3 | -10.666 | | 9.111 | | .725 |  |
|  | |  |  |  |  | 3=25 | 3=68.24 | 2-3 | -0.676 | | 8.878 | | 1.000 |  |
| Mental well-being | |  |  |  |  |  |  |  |  | |  | |  |  |
| Concerns about falling (n=169) | | Income | 6.500 | 4 | .165 | 1=30 | 1=92.17 | 1-2 | -11.521 | | 12.983 | | 1.000 |  |
|  | |  |  |  |  | 2=24 | 2=103.69 | 1-3 | -8.202 | | 12.457 | | 1.000 |  |
|  | |  |  |  |  | 3=28 | 3=83.96 | 1-4 | -14.722 | | 10.302 | | 1.000 |  |
|  | |  |  |  |  | 4=72 | 4=77.44 | 1-5 | -13.200 | | 14.991 | | 1.000 |  |
|  | |  |  |  |  | 5=15 | 5=78.97 | 2-3 | -19.723 | | 13.187 | | 1.000 |  |
|  | |  |  |  |  |  |  | 2-4 | -26.243 | | 11.174 | | .188 |  |
|  | |  |  |  |  |  |  | 2-5 | -24.721 | | 15.603 | | 1.000 |  |
|  | |  |  |  |  |  |  | 3-4 | -6.520 | | 10.558 | | 1.000 |  |
|  | |  |  |  |  |  |  | 3-5 | -4.998 | | 15.169 | | 1.000 |  |
|  | |  |  |  |  |  |  | 4-5 | -1.522 | | 13.455 | | 1.000 |  |
|  | | Education | 2.326 | 2 | .313 | 1=66 | 1=91.23 | 1-2 | -8.214 | | 8.052 | | .923 |  |
|  | |  |  |  |  | 2=73 | 2=83.02 | 1-3 | -15.135 | | 10.439 | | .441 |  |
|  | |  |  |  |  | 3=30 | 3=76.10 | 2-3 | -6.921 | | 10.281 | | 1.000 |  |
| Health-related quality of life (HrQoL, n=172) | | Income | 1.323 | 4 | .858 | 1=30 | 1=82.53 | 1-2 | -7.587 | | 13.169 | | 1.000 |  |
|  | |  |  |  |  | 2=25 | 2=90.12 | 1-3 | -0.068 | | 12.664 | | 1.000 |  |
|  | |  |  |  |  | 3=29 | 3=82.47 | 1-4 | -7.563 | | 10.546 | | 1.000 |  |
|  | |  |  |  |  | 4=73 | 4=90.10 | 1-5 | -3.833 | | 15.378 | | 1.000 |  |
|  | |  |  |  |  | 5=15 | 5=78.70 | 2-3 | -7.654 | | 13.272 | | 1.000 |  |
|  | |  |  |  |  |  |  | 2-4 | -0.024 | | 11.269 | | 1.000 |  |
|  | |  |  |  |  |  |  | 2-5 | -11.420 | | 15.883 | | 1.000 |  |
|  | |  |  |  |  |  |  | 3-4 | -7.630 | | 10.675 | | 1.000 |  |
|  | |  |  |  |  |  |  | 3-5 | -3.766 | | 15.466 | | 1.000 |  |
|  | |  |  |  |  |  |  | 4-5 | -11.396 | | 13.786 | | 1.000 |  |
|  | | Education | 1.663 | 2 | .435 | 1=68 | 1=88.23 | 1-2 | -0.887 | | 8.169 | | 1.000 |  |
|  | |  |  |  |  | 2=74 | 2=89.11 | 1-3 | -12.095 | | 10.526 | | .652 |  |
|  | |  |  |  |  | 3=30 | 3=76.13 | 2-3 | -12.982 | | 10.659 | | .769 |  |
| HrQoL – visual analogue scale (HrQoL VAS, n=171) | | Income | 2.495 | 4 | .646 | 1=29 | 1=77.16 | 1-2 | -4.025 | | 13.402 | | 1.000 |  |
|  | |  |  |  |  | 2=25 | 2=81.18 | 1-3 | -15.207 | | 12.896 | | 1.000 |  |
|  | |  |  |  |  | 3=29 | 3=92.36 | 1-4 | -12.948 | | 10.779 | | 1.000 |  |
|  | |  |  |  |  | 4=73 | 4=90.10 | 1-5 | -1.711 | | 15.618 | | 1.000 |  |
|  | |  |  |  |  | 5=15 | 5=78.87 | 2-3 | -11.182 | | 13.402 | | 1.000 |  |
|  | |  |  |  |  |  |  | 2-4 | -8.923 | | 11.379 | | 1.000 |  |
|  | |  |  |  |  |  |  | 2-5 | -2.313 | | 16.038 | | 1.000 |  |
|  | |  |  |  |  |  |  | 3-4 | -2.259 | | 10.779 | | 1.000 |  |
|  | |  |  |  |  |  |  | 3-5 | -13.495 | | 15.618 | | 1.000 |  |
|  | |  |  |  |  |  |  | 4-5 | -11.236 | | 13.921 | | 1.000 |  |
|  | | Education | 0.022 | 2 | .989 | 1=67 | 1=86.06 | 1-2 | -0.352 | | 8.281 | | 1.000 |  |
|  | |  |  |  |  | 2=74 | 2=86.41 | 1-3 | -1.210 | | 10.788 | | 1.000 |  |
|  | |  |  |  |  | 3=30 | 3=84.85 | 2-3 | -1.562 | | 10.629 | | 1.000 |  |
|  | |  |  |  |  |  |  |  |  | |  | |  |  |
|  | |  | [**χ²**](https://statistikguru.de/spss/chi-quadrat-test-unabhaengigkeit/einleitung-6.html) | **Degrees of freedom** | **p-value** | **n** | **/** | **Pairwise Comparison** | **Test Statistics** | | **/** | | **Adjusted signi-ficance** |  |
| Health care utilization | |  |  |  |  |  |  |  |  | |  | |  |  |
| Follow-up physician visits (n=172) | | Income | [χ²](https://statistikguru.de/spss/chi-quadrat-test-unabhaengigkeit/einleitung-6.html) (3.710) | 4 | .447 | 1=30  2=25  3=29  4=73  5=15 |  | 1-2  1-3  1-4  1-5  2-3  2-4  2-5  3-4  3-5  4-5 | 0.742  0.023  0.647  0.402  0.506  3.007  1.778  0.947  0.570  0.008 | |  | | 1.000  1.000  1.000  1.000  1.000  1.000  1.000  0.331  1.000  1.000 |  |
|  | | Education | [χ²](https://statistikguru.de/spss/chi-quadrat-test-unabhaengigkeit/einleitung-6.html) (6.638) | 2 | **.036*** | 1=68  2=74  3=30 |  | 1-2  1-3  2-3 | 0.110  0.493  0.922 | |  | | 1.000  0.118  **0.032*** |  |
| Therapy appointments (n=172) | | Income | χ² (0.737) | 4 | .947 | 1=30  2=25  3=29  4=73  5=15 |  | 1-2  1-3  1-4  1-5  2-3  2-4  2-5  3-4  3-5  4-5 | 0.465  0.010  0.136  0.048  0.596  0.205  0.127  0.235  0.090  0.001 | |  | | 1.000  1.000  1.000  1.000  1.000  1.000  1.000  1.000  1.000  1.000 |  |
|  | | Education | [χ²](https://statistikguru.de/spss/chi-quadrat-test-unabhaengigkeit/einleitung-6.html) (0.922) | 2 | .631 | 1=68  2=74  3=30 |  | 1-2  1-3  2-3 | 0.110  0.493  0.922 | |  | | 1.000  1.000  1.000 |  |
|  | |  | **Test Statistics (H)** | **Degrees of freedom** | **p-value** | **n** | **Mean rank** | **Pairwise Comparison** | **Test Statistics (H)** | | **Standard deviation** | | **Adjusted signi-ficance** |  |
| Characteristics | |  |  |  |  |  |  |  |  | |  | |  |  |
| Age (n=172) | | Income | 0.595 | 4 | .964 | 1=30 | 1=81.28 | 1-2 | -7.333 | | 13.474 | | 1.000 |  |
|  | |  |  |  |  | 2=25 | 2=88.60 | 1-3 | -8.716 | | 12.957 | | 1.000 |  |
|  | |  |  |  |  | 3=29 | 3=89.98 | 1-4 | -5.939 | | 10.790 | | 1.000 |  |
|  | |  |  |  |  | 4=73 | 4=87.21 | 1-5 | -2.033 | | 15.734 | | 1.000 |  |
|  | |  |  |  |  | 5=15 | 5=83.21 | 2-3 | -1.383 | | 13.579 | | 1.000 |  |
|  | |  |  |  |  |  |  | 2-4 | -1.395 | | 11.530 | | 1.000 |  |
|  | |  |  |  |  |  |  | 2-5 | -5.300 | | 16.250 | | 1.000 |  |
|  | |  |  |  |  |  |  | 3-4 | -2.777 | | -10.922 | | 1.000 |  |
|  | |  |  |  |  |  |  | 3-5 | -6.683 | | 15.824 | | 1.000 |  |
|  | |  |  |  |  |  |  | 4-5 | -3.905 | | 14.105 | | 1.000 |  |
|  | | Education | 21.418 | 2 | .**001**** | 1=68 | 1=108.21 | 1-2 | -35.977 | | 8.358 | | 1.000 |  |
|  | |  |  |  |  | 2=74 | 2=72.24 | 1-3 | -35.747 | | 10.905 | | **.000**** |  |
|  | |  |  |  |  | 3=30 | 3=72.47 | 2-3 | -0.230 | | 10.7969 | | **.003**** |  |
|  | |  | [**χ²**](https://statistikguru.de/spss/chi-quadrat-test-unabhaengigkeit/einleitung-6.html) | **Degrees of freedom** | **p-value** | **n** | **/** | **Pairwise Comparison** | **Test Statistics** | | **/** | | **Adjusted signi-ficance** |  |
| Sex (n=172) | | Income | [χ²](https://statistikguru.de/spss/chi-quadrat-test-unabhaengigkeit/einleitung-6.html) (10.463) | 4 | **.033*** | 1=30  2=25  3=29  4=73  5=15 |  | 1-2  1-3  1-4  1-5  2-3  2-4  2-5  3-4  3-5  4-5 | 2.554  2.046  0.001  1.607  8.295  3.198  6.593  2.993  0.008  2.085 | |  | | 1.000  1.000  1.000  1.000  **0.039***  0.737  0.102  0.836  1.000  1.000 |  |
|  | | Education | [χ²](https://statistikguru.de/spss/chi-quadrat-test-unabhaengigkeit/einleitung-6.html) (1.238) | 2 | .539 | 1=68  2=74  3=30 |  | 1-2  1-3  2-3 | 0.899  0.852  0.037 | |  | | 1.000  1.000  1.000 |  |

Note. ADLs:0-100 points, cognitive status:0-30 points, physical performance: 0-12 points, concerns about falling: 7-28 points, HrQoL: -0.205-0.999 points, HrQoL-VAS:0-100 percent, p-values were adjusted with Bonferroni-correction. * p <.05. ** p<.01.

**Table S2: Correlation coefficients for SES, health outcomes, covariates, and occupation.**

| Variable | n | M | SD | 1 | 2 | 3 | 4 | 5 | 6 | 7 | 8 | 9 | 10 | 11 | 12 | | 13 | | 14 | | 15 | | 16 | |  |
| --- | --- | --- | --- | --- | --- | --- | --- | --- | --- | --- | --- | --- | --- | --- | --- | --- | --- | --- | --- | --- | --- | --- | --- | --- | --- |
| SES |  |  |  |  |  |  |  |  |  |  |  |  |  |  |  | |  | |  | |  | |  | |  |
| 1. Income | 172 | 2238.61 | 1770.81 | - |  |  |  |  |  |  |  |  |  |  |  | |  | |  | |  | |  | |  |
| 2. Education | 172 | 10.28 | 1.92 | .328** | - |  |  |  |  |  |  |  |  |  |  | |  | |  | |  | |  | |  |
| Mental and functional performance |  |  |  |  |  |  |  |  |  |  |  |  |  |  |  | |  | |  | |  | |  | |  |
| 3. Activities of Daily Living (ADLs) | 170 | 95.44 | 10.49 | **.172*** | **.297**** | - |  |  |  |  |  |  |  |  |  | |  | |  | |  | |  | |  |
| 4. Cognitive status | 170 | 23.78 | 3.891 | **.200**** | **.331**** | **.298**** | - |  |  |  |  |  |  |  |  | |  | |  | |  | |  | |  |
| 5. Hand grip strength | 154 | 30.88 | 12,17 | .146 | **.356**** | **.488*** | **.184*** | - |  |  |  |  |  |  |  | |  | |  | |  | |  | |  |
| 6. Physical performance | 154 | 9.23 | 2.46 | **.285**** | **.276**** | **.389**** | **.356**** | **.272**** | - |  |  |  |  |  |  | |  | |  | |  | |  | |  |
| Physical activity |  |  |  |  |  |  |  |  |  |  |  |  |  |  |  | |  | |  | |  | |  | |  |
| 7. Step count | 128 | 952.66 | 551.46 | .069 | .062 | **.331**** | .092 | .094 | **.472**** | - |  |  |  |  |  | |  | |  | |  | |  | |  |
| 8. Sedentary time | 127 | 655.75 | 146.69 | .129 | .156 | **-.228*** | .029 | .028 | -.114 | **-.578**** | - |  |  |  |  | |  | |  | |  | |  | |  |
| Mental well-being |  |  |  |  |  |  |  |  |  |  |  |  |  |  |  | |  | |  | |  | |  | |  |
| 9. Concerns about falling | 169 | 10.02 | 4.23 | -.180* | -.120 | **-.465**** | **-.152*** | **-.405**** | **-.325**** | **-.253**** | .131 | - |  |  |  | |  | |  | |  | |  | |  |
| 10. Health-related quality of life (HrQoL) | 172 | 0.81 | 0.22 | -.011 | -.073 | **.364**** | .088 | .219** | .168* | .**291**** | -.166 | **-.384**** | - |  |  | |  | |  | |  | |  | |  |
| 11. Health-related quality of life visual analogue scale (HrQoL-VAS) | 171 | 65.95 | 21.65 | .053 | -.037 | **.264**** | .112 | **.184*** | **.293**** | **.299**** | -.149 | **-.356**** | **.498**** | - |  | |  | |  | |  | |  | |  |
| Health care utilization |  |  |  |  |  |  |  |  |  |  |  |  |  |  |  | |  | |  | |  | |  | |  |
| 12. follow-up physician visits | 172 | 0.46 | 0.50 | .106 | .088 | -.137 | .030 | -**.175*** | .074 | .006 | .049 | .086 | **-.186*** | -.016 | - | |  | |  | |  | |  | |  |
| 13. Therapy appointments | 172 | 0.66 | 0.47 | .060 | -.010 | .014 | .061 | -.078 | -.030 | .147 | -.127 | .043 | -.127 | -.087 | .065 | | - | |  | |  | |  | |  |
| Further characteristics |  |  |  |  |  |  |  |  |  |  |  |  |  |  |  | |  | |  | |  | |  | |  |
| 14.Age | 172 | 73.94 | 8.94 | -.031 | **-.338**** | **-.403**** | **-.488**** | **-.445**** | **-.434**** | **-.298**** | .075 | **.260**** | -.146 | **-.152*** | | .029 | | .021 | | - | |  | |  | |
| 15. Sex | 172 | 1.58 | 4.95 | -.063 | -.070 | -.126 | **-.182*** | **-.611**** | .018 | .084 | **-.251**** | .148 | -.092 | .067 | | .144 | | **.192*** | | -.027 | | - | |  | |
| 16.Occupation | 171 | 0.18 | 0.39 | -.013 | **.152*** | **.221**** | **.212**** | **.251**** | **.300**** | .159 | **-.182*** | -.114 | .084 | .090 | | -.051 | | -.070 | | **-.601**** | | .012 | | - | |

Note. ADLs:0-100 points, cognitive status:0-30 points, physical performance: 0-12 points, concerns about falling: 7-28 points, HrQoL: -0.205-0.999 points, HrQoL-VAS:0-100 percent, Spearman correlation coefficients were used, as data is not normally distributed. For correlations between two binary variables (physician visits, therapy appointments, sex, occupation) Phi coefficient was used. * p < .05. ** p<.01.

**Table S3: Results of regression analysis for health outcomes.**

|  |  |  | bootstrapped B-value | | 95% confidence interval | | bootstrapped standard error | | p-value | |  |
| --- | --- | --- | --- | --- | --- | --- | --- | --- | --- | --- | --- |
| Mental and functional performance | |  | |  | |  | |  | |  | |
| Activities of daily living (ADLs) ^a^ | | Intercept | | 77.950 | | [65.231, 87.949] | | 5.553 | | **<.001**** | |
| overall p-value= <.001**, adjusted R²=.088 | | Income | | 0.000 | | [0.000, 0.002] | | 0.001 | | .436 | |
|  | | Education | | 1.657 | | [0.853, 2.521] | | 0.511 | | **.005**** | |
| Cognitive status ^b^ | | Intercept | | 16.149 | | [13.157, 19.189] | | 1.696 | | **<.001**** | |
| overall p-value= <.001**, adjusted R²=.130 | | Income | | 0.000 | | [0.000, 0.001] | | 0.000 | | .186 | |
|  | | Education | | 0.692 | | [0.382, 0.952] | | 0.162 | | **<.001**** | |
| Hand grip strength ^c^ | | Intercept | | 8.134 | | [-0.215, 16.423] | | 4.430 | | .066 | |
| overall p-value= <.001**, adjusted R²=.110 | | Income | | 0.000 | | [-0.001, 0.003] | | 0.001 | | 0.328 | |
|  | | Education | | 2.288 | | [1.411, 3.057] | | 0.474 | | **<.001**** | |
| Physical performance ^d^ | | Intercept | | 5.759 | | [3.562, 7.708] | | 1.243 | | **<.001**** | |
| overall p-value= <.004**, adjusted R²=.059 | | Income | | 0.000 | | [0.000, 0.001] | | 0.000 | | .451 | |
|  | | Education | | 0.320 | | [0.045, 0.561] | | 0.129 | | **0.014*** | |
| Physical activity | |  | |  | |  | |  | |  | |
| Step count^e^ | | Intercept | | 5690.910 | | [1944.333, 9413.500] | | 1944.095 | | .006 | |
| overall p-value= .740 | | Income | | 0.111 | | [-0.503, 0.769] | | 0.261 | | .269 | |
|  | | Education | | 44.013 | | [-330.174, 410.973] | | 188.049 | | .817 | |
| Sedentary time ^f^ | | Intercept | | 543.559 | | [420.764, 640.461] | | 67.853 | | **<.001**** | |
| overall p-value= .277 | | Income | | -0.002 | | [-0.010, 0.050] | | 0.014 | | .756 | |
|  | | Education | | 11.110 | | [-1.930, 24.375] | | 6.906 | | .106 | |
| Mental well-being | |  | |  | |  | |  | |  | |
| Concerns about falling ^g^ | | Intercept | | 12.820 | | [9.611, 16.392] | | 1.729 | | **<.001**** | |
| overall p-value= .276 | | Income | | 0.000 | | [-0.001, 0.000] | | 0.000 | | .719 | |
|  | | Education | | -0.262 | | [-0.631, 0.157] | | 0.170 | | .125 | |
| Health-related quality of life (HrQoL) ^h^ | | Intercept | | 0.733 | | [0.540, 0.911] | | 0.097 | | <.001 | |
| overall p-value= .641 | | Income | | 0.000 | | [0.000, 0.000] | | 0.000 | | .501 | |
|  | | Education | | 0.007 | | [-0.010, 0.025] | | 0.009 | | .479 | |
| HrQoL – visual analogue scale (VAS) i | | Intercept | | 69.349 | | [49.587, 87.404] | | 9.457 | | **<.001**** | |
| overall p-value= .919 | | Income | | 0.000 | | [-0.002, 0.006] | | 0.001 | | .786 | |
|  | | Education | | -0.366 | | [-2.150, 1.304] | | 0.930 | | .693 | |
|  | |  | |  | | **95% confidence interval** | | **Odds ratio** | | **p-value** | |
| Health care utilization | |  | |  | |  | |  | |  | |
| Follow-up physician visits ^j^ | | Intercept | |  | |  | | 0.260 | | .341 | |
| overall p-value= .158 | | Income | |  | | [1.000, 1.000] | | 1.000 | | .114 | |
|  | | Education | |  | | [0.918, 1.281] | | 1.084 | | .297 | |
| Therapy appointments ^k^ | | Intercept | |  | |  | | 2.169 | | .383 | |
| overall p-value= .512 | | Income | |  | | [1.000, 1.000] | | 1.000 | | .348 | |
|  | | Education | |  | | [0.805, 1.141] | | 0.958 | | .632 | |

Note. ADLs: 0-100 points, cognitive status:0-30 points, physical performance: 0-12 points, concerns about falling: 7-28 points, HrQoL:-0.205-0.999 points, HrQoL-VAS:0-100 percent, a n= 169 for Model 1, b n= 169 for Model 1, c n=153 for Model 1, d n=153 for Model 1, e n=127 for Model 1, f n=126 for Model 1, g n=168 for Model 1, h n=171 for Model 1, i n=170 for Model 1, j n=172 for Model 1, k n=172 for Model 1. Different values in number of included persons (n) are due to missing values in predicting variables. *p<.05. **p<.01.

**Table S4: Comparison of regression results for model 2 of Activities of Daily Living** (**ADLs) including and excluding outliers.**

| Variable | Full sample ^a^ |  |  |  |  | Without outliers ^b^ | |  | | |  |  |
| --- | --- | --- | --- | --- | --- | --- | --- | --- | --- | --- | --- | --- |
|  | **Bootstrapped B-value** | **95% confidence interval** | **Boot-strapped standard error** | **p-value** | **Bootstrapped B-value** | | **95% confidence interval** | | **bootstrapped standard error** | **p-value** | | |
| Intercept | 114.345 | [102.104, 127.768] | 7.213 | **<.001**** | 106.331 | | [95.463, 117.310] | | 5.205 | **<.001**** | | |
| Income | 0.000 | [0.000, 0.003] | 0.001 | .518 | 0.000 | | [0.000, 0.003] | | 0.001 | .417 | | |
| Education | 1.029 | [0.311, 1.719] | 0.440 | **.028*** | 0.707 | | [0.220, 1.113] | | 0.286 | **.019*** | | |
| Age | -0.381 | [-0.583, -0.179] | 0.096 | **.003**** | -0.231 | | [-0.356, -0.124] | | 0.064 | **.002**** | | |
| Sex | -1.144 | [-3.947, 1.743] | 1.484 | .460 | -0.425 | | [-2.397, 1.870] | | 1.079 | .681 | | |

Note. ADLs:0-100 points, ^a^ n=169, overall p-value= <.001, adjusted R^2^=.174. ^b^ n=165, overall p-value=<.001, adjusted R^2^=.136. * p < .05. ** p<.01.

**Table S5: Comparison of regression results for model 2 of Activities of Daily Living (ADLs) including and excluding persons with occupation.**

| Variable | Full sample ^a^ |  |  |  | Retirees ^b^ |  | |  |  | | |
| --- | --- | --- | --- | --- | --- | --- | --- | --- | --- | --- | --- |
|  | **Bootstrapped B-value** | **95% confidence interval** | **bootstrapped standard error** | **p-value** | **Bootstrapped B-value** | | **95% confidence interval** | | | **bootstrapped standard error** | **p-value** |
| Intercept | 114.345 | [102.104, 127.768] | 7.213 | **<.001**** | 114.870 | | [97.096, 133.731] | | | 9.900 | **<.001**** |
| Income | 0.000 | [0.000, 0.003] | 0.001 | .518 | 0.000 | | [-0.001, 0.003] | | | 0.001 | .746 |
| Education | 1.029 | [0.311, 1.719] | 0.440 | **.028*** | 1.388 | | [0.571, 2.205] | | | 0.503 | **.013*** |
| Age | -0.381 | [-0.583, -0.179] | 0.096 | **.003**** | -0.416 | | [-0.681, -0.164] | | | 0.126 | **.008**** |
| Sex | -1.144 | [-3.947, 1.743] | 1.484 | .460 | -1.715 | | [-5.091, 1.660] | | | 1.760 | .342 |

Note. ADLs: 0-100 points, ^a^ n=169, overall p-value= <.001, adjusted R^2^=.174. ^b^ n=137, overall p-value=<.001, adjusted R^2^=.171. * p < .05. ** p<.01.

**Table S6: Comparison of regression results for model 2 of Activities of Daily Living (ADLs)** **across different age groups.**

| Variable | 60-69 years old ^a^ |  | |  |  | 70-79  years old ^b^ |  |  | | |  | 80 years and older ^c^ | |  | | |  | | |
| --- | --- | --- | --- | --- | --- | --- | --- | --- | --- | --- | --- | --- | --- | --- | --- | --- | --- | --- | --- |
|  | **Boot-strapped B-value** | | **95% con-fidence interval** | **Boot-strapped standard error** | **p-value** | **Boot-strapped B-value** | **95% confidence interval** | | **Boot-strapped standard error** | **p-value** | | **Bootstrapped B-value** | **95% confidence interval** | | **Boot-strapped standard error** | | | **p-value** |  |
| Intercept | 115.405 | [100.305, 135.133] | | 9.528 | **<.001**** | 135.839 | [81.707, 228.199] | | 34.660 | **.006**** | | 215.733 | [122.525, 320.938] | | | 52.149 | | **.002**** |  |
| Income | 0.000 | [-0.001, 0.001] | | 0.000 | .746 | 0.002 | [-0.001, 0.004] | | 0.001 | .241 | | 0.002 | [-0.002, 0.005] | | | 0.002 | | .338 |  |
| Education | 0.227 | [-0.706, 1.072] | | 0.444 | .623 | 0.585 | [-0.446, 2.356] | | 0.652 | .347 | | 1.379 | [-0.092, 2.983] | | | 0.887 | | .143 |  |
| Age | -0.282 | [-0.699, 0.057] | | 0.181 | .142 | -0.618 | [-1.832, 0.104] | | 0.493 | .192 | | -1.647 | [-2.974, -0.453] | | | 0.609 | | **.021*** |  |
| Sex | -0.781 | [-3.643, 1.888] | | 1.339 | .559 | -2.197 | [-7.475, 2.532] | | 2.563 | .405 | | -1.390 | [-7.999, 4.799] | | | 3.374 | | .686 |  |

Note. ADLs: 0-100 points, ^a^ n=64, overall p-value= .344, adjusted R^2^=.009. ^b^ n=48, overall p-value=.369, adjusted R^2^=.008, ^c^ n=55, overall p-value=.001, adjusted R^2^=.251, * p < .05. ** p<0.01.

**Table S7: Comparison of regression results for different covariates for Activities of Daily Living.**

| Variable | Model with age as covariate ^a^ | | | | | Model with age and education as variables ^b^ | | | | Model with age, sex and education as variables ^c^ | | | | | |
| --- | --- | --- | --- | --- | --- | --- | --- | --- | --- | --- | --- | --- | --- | --- | --- |
|  | **Boot-strapped B-value** | | **95% con-fidence interval** | **Boot-strapped standard error** | **p-value** | **Boot-strapped B-value** | **95% confidence interval** | **Boot-strapped standard error** | **p-value** | **Bootstrapped B-value** | **95% confidence interval** | **Boot-strapped standard error** | | **p-value** |  |
| Intercept | 129.416 | [114.983, 144.380] | | 7.663 | **<.001**** | 112.104 | [99.480, 125.949] | 6.528 | **<.001**** | 114.283 | [101.029, 128.946] | | 7.199 | **<.001**** |  |
| Age | -0.460 | [-0.681, -0.253] | | 0.111 | **<.001**** | -0.379 | [-0.570, -0.204] | 0.095 | **.003**** | -0.381 | [-0.572, -0.198] | | 0.095 | **.003**** |  |
| Sex |  |  | |  |  |  |  |  |  | -1.116 | [-4.000,  1.741] | | 1.482 | .474 |  |
| Education |  |  | |  |  | 1.105 | [0.372, 1.939] | 0.399 | **0.012*** | 1.082 | [0.339,  1.864] | | 0.406 | **.016*** |  |

Note. ADLs: 0-100 points, ^a^ overall p-value= <.001, adjusted R^2^=.149. ^c^ overall p-value=<.001, adjusted R^2^=.180, ^c^ overall p-value=<.001, adjusted R^2^=.178, * p < .05. ** p<0.01.
